# Frustrated Teachers: An REBT Intervention to Reduce Frustration Discomfort in Middle School Teachers—A Quasi-Experimental Study

**DOI:** 10.3390/ejihpe15040057

**Published:** 2025-04-08

**Authors:** Claudia Lupuleac, Darian Faur, Florin Alin Sava

**Affiliations:** Department of Psychology, West University of Timisoara, Vasile Parvan 4, 300223 Timisoara, Romania; claudia.denes69@e-uvt.ro (C.L.); darian.faur99@e-uvt.ro (D.F.)

**Keywords:** middle school teachers, discomfort to frustration, irrational beliefs, pupil control ideology, Rational Emotive Behavioral Therapy

## Abstract

Teaching requires sustained emotional effort from educators, and the gap between reality and teachers’ expectations regarding student engagement can negatively affect their psychological well-being. The current research implemented a program to reduce their frustration discomfort. The main objective was to investigate the effectiveness of an intervention program based on the Rational-Emotive Behavioral Therapy (REBT) framework, specifically designed for middle school teachers. A sample of 54 teachers was gathered and conveniently divided into two equal groups: intervention and control. Participants in the intervention group participated in a weekly REBT group intervention program for six weeks, while the control group did not receive any intervention. Evaluations were conducted at four points: pretest, post-test, and follow-up assessments three and seven months after the intervention. Frustration discomfort was the primary dependent variable, while low frustration tolerance as an irrational belief, and pupil control ideology were treated as secondary dependent variables. The results indicated that the intervention effectively reduced frustration discomfort, and this positive effect was maintained during the follow-up assessments.

## 1. Introduction

Nowadays, teachers are often impacted by the uncertainty surrounding reforms in the public education system, the heavy administrative workload, students’ lack of interest in the subjects being taught, inappropriate behavior in the classroom or school environment, and competition between classes and schools (see Panisoara et al., 2020; Ciuhan et al., 2022). These obstacles can lead to frustration, which may, over time, manifest as various emotional and behavioral issues (Guo et al., 2024). Research in educational psychology has emphasized the significance of the frustration that teachers experience and its impact on education. Frustration causes emotional disturbances in teachers and can directly affect their students. Constant displays of frustration from teachers can influence students’ academic motivation, behavior related to education, and school goals (Park & Ramirez, 2022). Because frustration is often at the root of these outcomes, it is essential to highlight potential underlying mechanisms that could explain it in educational settings.

### 1.1. Irrational Beliefs Among Teachers: Low Frustration Tolerance and Student Control Ideology

There are multiple frameworks that could explain the challenges mentioned above. One of them is the transactional model of stress (Lazarus & Folkman, 1987), which was applied in the educational context to emphasize that teachers’ stress can be viewed as a response to the perception of stressors (such as unmotivated students, time pressures and workloads, conflicts with administrations) and the individual’s available resources to deal with the stressors (Bernard, 2016). The gap between the teacher’s assessment of the stressor and the personal resources available to cope with it often creates a conducive environment for irrational beliefs (Huk et al., 2019).

Another framework is the Rational-Emotive Behavioral Therapy (REBT), proposed by Ellis (1962), which posits that disturbances in the thought process can explain the maladaptive emotional responses that individuals exhibit when facing stressful situations. More specifically, evaluations that are not congruent with reality (irrational statements) are at the root of the distress experienced by these individuals (DiGiuseppe et al., 2013). It has been shown that high levels of irrational beliefs in teachers are linked with stress, psychological hardiness, and low self-efficacy (Popov et al., 2015; Bernard, 2016). Moreover, irrational beliefs in teachers mediate the relationship between self-efficacy and innovative work behavior, ultimately impacting the teacher’s ability to establish complex and non-linear educational classroom activities (Gkontelos et al., 2023).

Among the specific themes that define irrationality in teachers are demandingness (“Students must do exactly as I say when I’m in class”), self-downing (“I’m awful at what I’m doing”), and low-frustration tolerance statements (“I can’t stand when the student interrupts me when I talk”) (Warren, 2013; C. H. Bora et al., 2013). Low frustration tolerance is a central irrational thought in REBT, causing individuals to experience emotional distress even when dealing with minor stressors (Huk et al., 2019). Frustration tolerance is also linked with academic outcomes: high tolerance for frustration is positively correlated with academic performance in teachers (Shi et al., 2021). Additionally, teachers with a higher tolerance for frustration tend to adopt a more positive and constructive attitude when faced with unpleasant school events, leading to greater job satisfaction (Park & Ramirez, 2022). Harrington (2005, 2006) expands the concept of frustration intolerance as a more nuanced construct that helps to distinguish among various ways people react to frustration and that helps to differentiate between different sources of frustration (e.g., achievement, entitlement, emotional intolerance, discomfort intolerance).

Teachers’ tendency to think irrationally and, more specifically, to engage in low frustration tolerance thinking regarding their work also impacts the quality of the relationship established with their students. The pupil control ideology is an important concept that influences the teacher-student relationship. It defines whether the teacher will display a custodial view over the student’s behavior (by considering misbehaving as a sign of disrespect toward the teacher and the appeal to authority and strict order to counterbalance it) or a humanistic view (which emphasizes that misbehaving is linked with unmet student needs, which eventually require to be nurtured) (Samfira & Sava, 2023). Prior research has shown that teachers who adopt a custodial view tend to display higher levels of irrational beliefs, perfectionism, and maladaptive cognitive schemas of mistrust and entitlement (Samfira & Sava, 2021). On the other hand, the humanistic approach is correlated with the development of soft skills in students that extends beyond school performance, pleading for long-term benefits and competence development instead of mere knowledge gain (Ding & Wang, 2018).

### 1.2. REBT-Based Interventions in Educational Settings

The first REBT applications for teachers were developed by Ellis (1962) as a rational education program aimed at managing the irrational beliefs responsible for occupational discomfort. An extension of REBT in the educational context was proposed by W. Knaus (1977) under the form of Rational Emotive Education (REE) or Rational Emotive Behavior Education (REBE). This intervention is based on the ABC theory of emotions proposed by Ellis (1962), which offers a structured framework to conceptualize the REBT strategies: an activating event, such as a student interrupting the class (A), leads to a cognitive interpretation that can be irrational—“I can’t stand when the student does that” (B), which in turn elicits a dysfunctional emotional response, such as anger (C). REBT and its derivate programs are well-supported interventions in dealing with irrational beliefs in educational settings (see David et al. (2018) for an extended meta-analysis on REBT and Trip et al. (2007) for an older meta-analysis on REE).

Most studies involving REBT-based interventions in education settings focused on modifying irrational beliefs (irrespective of the main theme) as the primary outcome. Studies of the utility of REBT principles in various educational settings have reported an improved control of teachers’ emotional health regardless of students’ maladaptive behaviors by addressing the irrational beliefs identified in their thinking patterns (Maag, 2008). C. H. Bora et al. (2013) implemented an REBT-based intervention program (REBE) on 40 Romanian teachers. The study’s primary objective was that, by the end of the three-month REBE intervention, teachers could use cognitive restructuring techniques to address dysfunctional beliefs and associated inferences, thereby improving their emotional well-being. The results demonstrated the mediating effect between changing irrational beliefs and teachers’ emotional and behavioral changes, confirming the validity of REBE interventions in the educational field.

Similarly, Caruso et al. (2018) implemented a REBE intervention focusing on primary school students and teachers. The intervention for students was based on storytelling. In contrast, the intervention for teachers was focused on the ABC framework, thought process monitoring, and cognitive restructuring (formulating rational alternatives to the irrational beliefs identified). As an outcome of the intervention, students revealed an improved tendency toward rational thinking, while teachers displayed higher levels of perceived self-efficacy.

REBT-based interventions were effectively utilized to reduce negative emotional responses stemming from irrational thinking in high-stress educational environments. As a result of increased rational thinking, levels of discomfort and stress among special education teachers were reduced (Onuigbo et al., 2018). Similarly, emotional distress and job-related burnout were significantly reduced in teachers working in the same setting as previously mentioned (Ugwoke et al., 2018).

The literature regarding the REBT interventions in school settings that modify teachers’ control ideology over students is scarce. To the best of our knowledge, only one study implemented a program based on REBT principles to diminish the tendency to control the students in a custodial manner—the results indicated a tendency to improve control ideology, shown by lower custodial ideology means from pretest to post-test; however, the overall difference was not significant (Samfira & Sava, 2023). Similarly, while studies that focus on REBT in school settings tend to measure irrational beliefs globally, they fail to address specific patterns of irrationality, such as designing REBT interventions for demandingness, self-downing, or low frustration tolerance.

### 1.3. The Present Study

The current research aims to address a gap in the literature by focusing on beliefs related to low frustration tolerance while also accounting for the student control ideology. Therefore, our main hypothesis is that there is a significant decrease in frustration discomfort for participants in the intervention group in comparison with participants in the control group (H1), followed by a complementary one that there is a significant reduction of low frustration tolerance irrational beliefs in the intervention group in comparison with the control group (H2).

Additionally, this study examines the effectiveness of REBT strategies in indirectly modifying teachers’ custodial control ideology regarding their students. Hence, our third hypothesis is that there is a significant improvement in the humanistic ideology of pupil control for participants in the experimental group (H3). Finally, we integrated these assumptions into a single model and hypothesized that frustration tolerance would mediate the effects of the intervention on the student control ideology (H4).

To our knowledge, this is the first study to link specific irrational beliefs (i.e., low frustration tolerance) to pupil control ideology in middle school teachers and address it with an REBT-based intervention. Investigating these aspects brings added value to the literature. First, as already explained, teachers are often exposed to frustrating situations, and testing the effectiveness of six-session group interventions tailored to this common problem has practical relevance. The intervention utility increases if spill-over effects are seen, so that working on frustration discomfort leads to positive effects on the general well-being of teachers due to a lower extent of low frustration irrational beliefs, and also leads to a better school climate, as facilitating a humanistic pupil-control ideology, which is highly predictive for positive student-teacher interactions.

## 2. Materials and Methods

### 2.1. Participants and Procedure

The research sample consisted of 54 middle school teachers who were non-randomly assigned to two experimental conditions: intervention vs. control group. The lack of randomization was due to accessibility concerns. A cluster sampling method was chosen, and participants were selected based on their primary year of middle school teaching and their baseline level of frustration. The average age of participants was 46 years, with an average teaching experience of 25 years. Of these, 82.7% were female, 92% held tenured teaching positions, 72% had the highest teaching qualification (Grade I), 17% had Grade II, 9% had obtained a permanent certification, and 2% were beginners. The main inclusion criterion was that the participant needed to be a middle school teacher (irrespective of the years of experience) and that they had at least an average score at screening (pretest) on the frustration discomfort measure. The recruitment method used self-selection, and the sampling method was based on the primary year of middle school in which they were teaching (cluster-assigned). All participants were teachers residing and teaching in small urban areas in the central-western part of Romania.

Based on our convenience sample, a sensitivity analysis was conducted using G*Power (Faul et al., 2007). The analysis indicated that with 80% power and a standard significance level of α = 0.05, our sample size of N = 54 can detect a minimum effect size of f = 0.46 and a Cohen’s d = 0.92. Although this suggests a large effect, previous studies have reported even larger effect sizes for an REBT-based intervention to improve teachers’ low frustration tolerance (C. H. Bora et al., 2013).

To prevent the risk of non-equivalence between groups due to selection bias, which could negatively impact the internal validity of the study results, participants were allocated to the two groups based on a statistical analysis of the total frustration discomfort measure (*t*-test). This ensured that there were no significant differences in the dependent variable (frustration tolerance level) between the two groups at the pre-intervention stage. The results are presented in Table 1.

Before assignment to the two groups, teachers completed an online informed consent form for participation in the study. The intervention lasted six weeks and was conducted online. Pretest and post-test data were collected for both experimental and control groups, followed by a 3-month and a 7-month follow-up. Participants in the study (both the intervention and control groups) were not financially compensated, and their participation was voluntary. The first author was involved in intervention administration and outcome assessment; hence, no blinding was involved. TREND reporting guidelines for nonrandomized/quasi-experimental study designs were used for this study (Haynes et al., 2021).

### 2.2. Measures

Frustration Discomfort Scale (FDS). Developed by Harrington (2005), it consists of 28 items designed to evaluate irrational evaluative beliefs that individuals may hold when encountering frustrating or discomforting situations. Participants respond using a 5-point Likert scale (1 = absent, 5 = very strong). The scale comprises four dimensions that can be summed up in a total score: (1) Discomfort Intolerance (“I cannot stand the idea of performing overly difficult tasks.”), (2) Inequity/Unfairness Intolerance (“I cannot stand people who interfere with my desires.”), (3) Emotional Distress Intolerance (“I cannot tolerate situations that might upset me!”), and (4) Intolerance of Goal-Frustration (“I cannot stand being prevented from reaching my full potential!”).

The reliability indices retrieved from the original study indicate that FDS has an adequate internal consistency (all dimensions α > 0.84; Harrington, 2005) and is a reliable tool for assessing frustration intolerance in individuals. FDS was chosen because it was previously employed in research regarding Romanian samples. The internal consistency coefficient for the whole instrument was α = 0.96, indicating that FDS is highly reliable in measuring discomfort to frustration in the Romanian population (Lupuleac & Sava, 2024). For our study, the internal consistency of the FDS was also high, α = 0.90.

Teachers’ Irrational Beliefs Scale (TIBS). The Teachers’ Irrational Beliefs Scale (TIBS) was initially developed by Bernard (1988) and later adapted for the Romanian population by C. Bora et al. (2009). The TIBS-RO (C. Bora et al., 2009) is a psychometric scale based on the principles of REBT. The scale consists of 20 items, each rated on a 5-point Likert scale (1 = strongly disagree, 5 = strongly agree). It measures various types of irrational beliefs teachers hold within the educational context. Unlike the original version, which evaluated four factors, the Romanian adaptation underwent confirmatory factor analysis, resulting in a three-factor solution (Absolute Demands on Others, Global Self-Evaluation, and Low Frustration Tolerance). Scores are calculated separately for each factor, with higher scores indicating higher irrational beliefs.

For this research, only the items measuring Low Frustration Tolerance were used, as this aspect is the primary focus of the study. While the original study reported an acceptable internal consistency index for this dimension (α = 0.77; Bernard, 1998), it was reported to be very low in the Romanian population (α = 0.44; C. Bora et al., 2009). The internal consistency of TIBS in our sample also proved to be weak, α = 0.56. Although TIBS has low internal consistency, it is a rather widely used tool for identifying irrationality among teachers in educational settings, which was central to our study.

Pupil Control Ideology Scale (PCI). Developed initially by Willower et al. (1967), PCI is a psychometric instrument designed to assess teachers’ perspectives on pupil control ideology in the school environment. The Romanian adaptation of PCI was validated by Sava (2002) and consists of 20 items. Respondents rate each statement on a 5-point Likert scale (1 = strongly disagree, 5 = strongly agree). Higher scores indicate a custodial ideology of student control, which is characterized by strict discipline, coercive measures, and punitive approaches. Lower scores suggest a humanistic ideology, emphasizing collaboration, understanding, and support for students. Most items are scored directly, except for items 5 and 13, which are reverse-scored. The original scale’s reliability is high, with Cronbach’s alpha values ranging from 0.80 to 0.91 (Willower et al., 1967). The coefficients are slightly lower for the Romanian sample (α = 0.74; Sava, 2002). Similar internal consistency indices were obtained for our study (α = 0.77). Since pupil control ideology was a specific outcome of our intervention, the PCI scale was the preferred option as it directly measures this construct and has been utilized in previous research involving the Romanian population.

### 2.3. Intervention

The intervention program, generically called “The Rational Teacher”, was theoretically and practically based on reference works in the field: (1) “The Practice of Rational Behavior Therapy, Second Edition” (Ellis & Dryden, 1997)—used for teaching group counseling principles and core REBT concepts. (2) “How to Conquer Your Frustrations” (W. J. Knaus, 1983), (3) “SOS Help for Emotions: Managing Anxiety, Anger, and Depression, Second Edition” (Clark, 2002)—providing training strategies for improving frustration tolerance and coping mechanisms in professional life. Once designed, the intervention was cross-validated by experts working in educational psychology to improve the intervention.

This program aimed to support a group of middle school teachers by enhancing their tolerance for frustration and discomfort in response to the complexity and unpredictability of modern daily life and frustrating situations in the workplace. The rationale behind this program was that an increase in discomfort tolerance (to frustration) due to flexible (rationale) interpretations of specific situations in the educational environment would also eventually lead to a humanistic ideology of student control. There were six sessions in total, spanning a 6-week period (each session lasting 75 min). All sessions were delivered online via Google Meet (the intervention program was implemented in 2021 during the COVID-19 pandemic lockdown). Confidentiality was assured by assigning a password to each participant who agreed to participate in the intervention. The content of each session is summarized in Table 2.

### 2.4. Analyses

To test the working hypotheses and measure the effect of REBT intervention on the primary dependent variable (the total score of frustration discomfort recorded on the FDS scale), as well as secondary variables (the total scores of irrational beliefs about frustration measured by the TIBS scale and the level of custodial ideology of student control measured by the PCI scale), a 2 × 4 mixed ANOVA technique was used. This approach is particularly suitable for the design (as long as the parametrical assumptions are met) because it accounts for the repeated measurements of the same dependent variables across multiple time points. Testing the intervention and control groups at pre-intervention, post-intervention, 3-month follow-up, and 7-month follow-up allows for a detailed analysis of how the intervention influences the primary (FDS) and secondary (TIBS and PCI) dependent variables over time. This method enables the detection of both between-subject effects (comparing the experimental and control groups) and within-subject effects (tracking changes within individuals), making it ideal for identifying changes and the long-term impact of the intervention. The analyses were carried out in SPSS v. 25.

**Figure 1:**
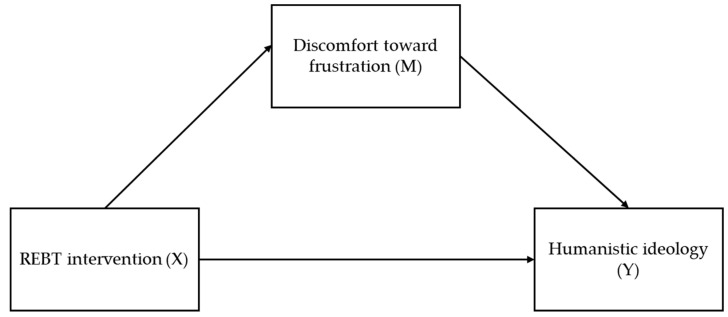
Conceptual diagram for the mediation model.

Regarding the mediation model, the conceptual model on which we based our analysis is presented in Figure 1.

For testing the mediation model, we used the PROCESS macro v. 4.2 for SPSS (Hayes, 2022). The mediation analysis was carried out through bootstrapping (Bootstrap = 5000, CI = 95%). We adopted the standard approach in testing mediation models proposed by Baron and Kenny (1986), where the following criteria are cumulatively established: (i) the independent variable is associated with the dependent variable; (ii) the mediator variable is associated with the dependent variable; (iii) the relationship between the independent variable (the intervention in our case) and the dependent variable (score change in pupil control ideology) are controlled by the mediator variable (score change in frustration to discomfort). The mediation method was chosen as a conservative approach to highlight the relationship between the REBT-based intervention and pupil control ideology, while controlling frustration discomfort as a mediator.

## 3. Results

Before proceeding with the main hypothesis testing, we analyzed the descriptive indices for the outcomes in all conditions (between-subject: grouping factor; within-subject: measurement time). As indicated in Table 3, the Skewness and Kurtosis indices are situated in the accepted interval (−2, +2) for the normality of the distribution assumption. The Shapiro-Wilk test was non-significant for most conditions, indicating that a parametric approach to the main model testing is favorable. The participants presented no missing data across the four measurement times; therefore, no imputation method or other methods for adjusting the parameters based on the pattern of missing data were required.

### 3.1. Primary Outcome: Discomfort Toward Frustration

The primary outcome, frustration discomfort, showed a significant interaction between group (intervention vs. control) and measurement time (pretest, post-test, 3-month follow-up, and 7-month follow-up), as indicated by the mixed ANOVA results, F(3, 156) = 13.56, *p* < 0.001, η^2^ = 0.207. Sphericity was assumed, Mauchly’s W (5) = 0.92, *p* = 0.52, suggesting that frustration tolerance (measured by FDS scores) evolved differently over time between the intervention and control groups.

The simple effects analysis (Bonferroni test; see Table 2 for means and standard deviations) showed no significant difference between the groups at baseline, t(52) = −0.85, *p* = 0.398. However, significant differences emerged at post-test, t(52) = 3.53, *p* < 0.001, 3-month follow-up, t(52) = 6.24, *p* < 0.001, and 7-month follow-up, t(52) = 2.76, *p* < 0.01.

**Figure 2:**
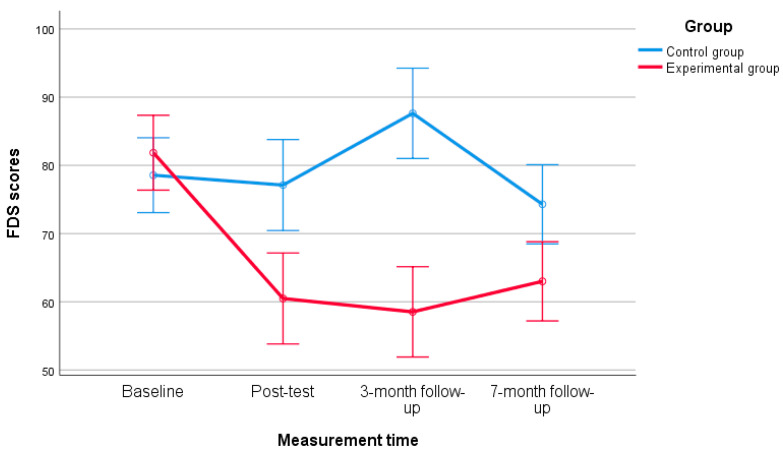
Mean evolution of the FDS (95% CI) between conditions across time.

Additionally, in the experimental group, FDS scores at post-test remained stable across both follow-ups, F(2, 52) = 0.733, *p* = 0.485, η^2^ = 0.027. Figure 2 illustrates the mean evolution of discomfort toward frustration for both groups over time.

These findings suggest that the REBT-based intervention effectively reduced teachers’ frustration discomfort, with sustained effects over time, as indicated by the significant difference between groups at the 7-month follow-up and the stability of FDS scores after the post-test in the experimental group.

### 3.2. Secondary Outcomes: Teachers’ Irrational Beliefs Toward Frustration and Control Ideology

Regarding teachers’ irrational beliefs toward frustration, the main interaction effect was marginal but non-significant, F(3, 156) = 2.54, *p* = 0.058, η^2^ = 0.047. While the between-person effect (group) was significant, F(1, 52) = 6.14, *p* < 0.01, η^2^ = 0.106, the within-person effect (measurement time) was not, F(3, 156) = 0.66, *p* = 0.575, η^2^ = 0.013. This suggests that, although the experimental group had lower average irrational belief scores than the control group (M = 16.20 vs. M = 18.03), irrationality toward frustration did not change significantly over time in either group.

A similar pattern was observed for teachers’ control ideology. The between-person effect was significant, F(1, 52) = 8.68, *p* < 0.01, η^2^ = 0.143, but the within-person effect was not, F(3, 156) = 1.16, *p* = 0.324, η^2^ = 0.022, nor was the interaction effect, F(3, 156) = 1.77, *p* = 0.154, η^2^ = 0.033. Although teachers in the experimental group exhibited a more humanistic control ideology on average (M = 53.06) compared to the control group (M = 58.84), this difference remained stable over time.

### 3.3. Analysis of the Mediation Model

We tested the mediation model based on the results obtained for the main model (and on the tendencies for interaction regarding the secondary outcomes). Here, we considered frustration discomfort as the mediator (M variable) that explains the interaction between the REBT intervention (X variable) and the outcome—pupil control ideology (Y variable). We first inspected the effects of X on M and then the effects of X on Y. The results indicated that both effects are significant: intervention on discomfort to frustration (ϐ = −0.440, t = −3.53, *p* < 0.001) and intervention on pupil control ideology (ϐ = −0.394, t = −3.08, *p* < 0.01). When both variables (X and M) are introduced in the model to predict the outcome, only the effect of discomfort to frustration is significant on pupil control ideology (ϐ = −0.453, t = −1.71, *p* = 0.093 for X → Y and ϐ = 0.375, t = 2.81, *p* < 0.01 for M → Y). This is consistent with the hypothesized model, namely that discomfort to frustration mediates between the effect of the intervention and the pupil control ideology.

For the total, direct, and indirect effects, bootstrapping with 5000 samples was used (CI = 95%) to test the significance of the mediation model. The coefficients are presented in Table 4.

Because the confidence interval includes 0 for the direct effect (LLCI = −9.57, ULCI = 0.756), the results indicate no significant direct effect of the intervention on pupil control ideology when the discomfort to frustration is controlled as a mediator. On the contrary, there is a significant indirect effect (LLCI = −7.04, ULCI = −0.390) of the intervention on pupil control ideology through the mediator variable, discomfort to frustration.

## 4. Discussion

The present study aimed to examine the effectiveness of an REBT-based intervention to decrease frustration discomfort in educational settings. The intervention also included outcomes regarding teachers’ irrationality toward frustration and their pupil control ideology. It was found that, regarding the discomfort to frustration, the intervention effectively reduced discomfort associated with frustration and increased frustration tolerance. Moreover, the intervention delivered stable results over time, captured in the difference between the experimental group and the control group at the 7-month follow-up and the stability of the FDS scores after the post-test in the experimental group.

These results are consistent with the literature regarding REBT-based interventions in educational settings. These interventions increase the perceived quality of work-life balance (Ogakwu et al., 2024), unconditional self-acceptance (Samfira & Sava, 2023), work value and ethical practices (Abiogu et al., 2021), self-efficacy (Caruso et al., 2018), while decreasing stress and irrational beliefs (Onuigbo et al., 2018; Popov et al., 2015; C. H. Bora et al., 2013) in teachers who are engaged in various educational settings, ranging from primary education to high school and special education. Moreover, the significant interaction effect observed between the group and time is primarily evident in the context of discomfort tolerance, which highlights a specific irrational theme—low frustration tolerance. This suggests that REBT interventions in school settings resemble practical strategies that can effectively target particular irrational thinking patterns.

Regarding the secondary outcomes, namely teachers’ irrational beliefs toward frustration and pupil control ideology, the main interaction was marginally non-significant for irrational beliefs facilitating low frustration tolerance and non-significant for the pupil-control ideology. Several potential explanations exist for inconclusive results related to teachers’ irrational beliefs toward frustration. First, it could be that our study was likely underpowered to capture the usually obtained effect sizes of REBT interventions on modifying irrational beliefs toward frustration. While our sample effectively captured the effect of the intervention on frustration discomfort, the effect size was not large enough to detect a significant effect for altering the irrational beliefs related to low frustration tolerance. Another possible explanation for this non-significant effect is the low internal consistency of the TIBS subscale that measures low frustration tolerance (e.g., a Cronbach’s alpha of 0.55 in our study for TIBS low frustration tolerance scale in comparison with a Cronbach’s alpha of 0.90 for the FDS scale). Since the TIBS scale was not reliable in assessing irrational responses to frustration, the variability in this outcome likely stemmed from measurement errors rather than actual differences over time within the experimental group. Another potential explanation for these divergent results is based on conceptual differences between frustration-related measurements. Whereas TIBS is a direct measure of irrational beliefs, FDS focuses on discomfort (the emotional experience of encountering frustration). It could be the case that the six-week group program is insufficient for altering beliefs, but it is appropriate to teach some coping strategies when encountering frustrating experiences. It is worth noticing that all above explanations could explain in an additive manner this marginal non-significant result, as this particular hypothesis could suffer both from measurement issues given the TIBS scale reliability issue as well as from an underpowered study given that our sensitivity analysis showed that the current study was adequately powered to detect only high effect sizes (d = 0.90). Given these limitations, it is less surprising that our findings differ from those obtained by C. H. Bora et al. (2013), who reported a significant decrease in teachers’ irrationality due to the REBT-based intervention.

As for the pupil control ideology, this finding is consistent with Samfira and Sava (2023). Of particular interest is that the mediation model we proposed for the present study was supported by data underlying a significant indirect effect of the intervention on the pupil control ideology via discomfort to frustration as a mediator. This effect indicates that, while our REBT-based intervention tailored to decrease frustration discomfort is effective, it could also indirectly influence the pupil control ideology, a concept that is a core predictor for the quality of teacher-pupil interaction (see Sava, 2002).

A final mention is warranted regarding the intervention’s online format. Previous studies examining programs designed to reduce teachers’ stress and delivered online have expressed concerns about participant engagement in these interventions (Lang et al., 2020). Despite these challenges, the online format provides promising results, indicating that teachers’ overall stress and burnout levels can be effectively countered with these programs (Ansley et al., 2021). Both emotional outcomes and regulatory processes, such as motivation, can be increased in teachers through online interventions (Daniels et al., 2021). While we acknowledge the challenge of effectively tracking participants’ engagement levels online, our current research yielded satisfactory results regarding reducing frustration discomfort in the experimental group. These positive outcomes were also sustained during the two follow-up evaluations. Thus, implementing online intervention programs to alleviate teachers’ frustration can be a viable alternative when face-to-face options are unavailable.

As already mentioned, the current study has several limitations. One limitation is the absence of randomization when assigning teachers to the two groups. While this is a common issue in educational intervention studies due to teachers’ busy schedules, it can introduce errors that may lead to invalid results, particularly if the baseline scores for the outcomes differ between the experimental conditions. Although this was not the case in the current research (see Table 1 for equivalence testing), ensuring the participants are randomly assigned to the experimental groups remains essential. A second limitation is the low sample size. Meta-analyses on REBT interventions (David et al., 2018; Trip et al., 2007) indicate that, generally, the observed effect size of the intervention on the outcomes is medium (medium-large in educational settings). While we obtained a large effect size for our primary outcome (similar to C. H. Bora et al., 2013), it is possible that, alongside the other explanations presented above, our study was underpowered for the secondary outcomes (since a sample size of N = 179 would have been required to obtain 80% power to detect a medium effect size Cohen’s f = 0.25 at a standard α = 0.05).

This research was one of the first to implement an REBT intervention for middle school teachers, focusing specifically on frustration-related discomfort. It evaluated the impact of this intervention on irrational beliefs toward frustration and pupil control ideology. The findings of this study have important implications for the development of teacher training programs. A concise training initiative grounded in REBT effectively alleviated feelings of discomfort linked to frustration among educators, resulting in noteworthy and lasting improvements. This suggests that such strategies can be seamlessly woven into the regular weekly meetings that teachers conduct to address various internal educational challenges.

On a different note, the results indicate that altering students’ control ideology is a more complex endeavor. This goal will likely necessitate multiple training sessions and a varied approach, perhaps one that emphasizes acceptance and commitment strategies. Future studies should further explore the connection between frustration tolerance and pupil control ideology by incorporating additional significant variables into their intervention programs while ensuring proper randomization and adequate sample size.

## Figures and Tables

**Table 1:** Data analysis regarding group equivalence.

Measured Variable	Total Sample (N = 54)	Experimental Group (N = 27)	Control Group (N = 27)	Equivalence Testing
Female	45 (83.3%)	24 (88.9%)	21 (77.8%)	X^2^(1) = 1.20; *p* = 0.273
Male	9 (16.7%)	3 (11.1%)	6 (22.2%)	
Age	M = 46.17 (SD = 7.68)	M = 47.59 (SD = 7.04)	M = 44.74 (SD = 8.16)	t(52) = −1.37, *p* = 0.175
Single	9 (16.7%)	5 (18.5%)	4 (14.8%)	X^2^(2) = 0.137, *p* = 0.934
Married	39 (72.2%)	19 (70.4%)	20 (74.1%)	
Divorced	6 (11.1%)	3 (11.1%)	3 (11.1%)	
Tenured	3 (5.6%)	26 (96.3%)	25 (92.6%)	X^2^(1) = 0.353, *p* = 0.552
Substitute	51 (94.4%)	1 (3.7%)	2 (7.4%)	
Years of experience	M = 23.80 (SD = 7.50)	M = 24.41 (SD = 7.40)	M = 23.19 (SD = 7.70)	t(52) = −0.594, *p* = 0.555
Permanent certification	3 (5.6%)	1 (3.7%)	2 (7,4%)	X^2^(2) = 1.04, *p* = 0.594
Grade II	8 (14.8%)	3 (11.1%)	5 (18.5%)	
Grade I	43 (79.6%)	23 (85.2%)	20 (74.1%)	
Frustration discomfort (FDS)—baseline	M = 80.20 (SD = 14.17)	M = 81.85 (SD = 14.93)	M = 78.56 (SD = 13.44)	t(52) = −0.852, *p* = 0.398

**Table 2:** Description of the sessions’ content.

Session Number	Description
Session 1	Introductory Exercises: clarification of specific group therapy rules, interactive mutual introductions, and assessment of teachers’ expectations from participating in this intervention program.
Session 2	Understanding the Basics REBT: introduction to the ABC model, teaching the relationship between thoughts—emotions—behavior.
Session 3	Understanding Low Frustration Tolerance: identifying irrational beliefs and underlying irrational thoughts, exploring different types of frustration intolerance, understanding the circular model of frustration intolerance, and identifying frustrating situations in the educational environment.
Session 4	Familiarization with Disputation Strategies: introduction to empirical, logical, and pragmatic disputation strategies, focus on semantic disputation of irrational beliefs related to low frustration tolerance (e.g., “I can’t stand this,” “I can’t tolerate it!”), understanding the ABCDE model.
Session 5	Alternative Strategies for Modifying Irrational Beliefs: transforming irrational beliefs about frustration into rational beliefs about tolerating discomfort, rational slogans, and rational-emotive imagery as cognitive restructuring techniques.
Session 6	Developing the Anti-Frustration Kit: using clarification questions to examine the empirical, logical, and semantic evidence of dysfunctional, irrational beliefs related to frustration, practicing relaxation exercises, such as square breathing, generating alternative solutions and rational slogans, and applying the “Triad of Rational Living” approach.

**Table 3:** Descriptive statistics for all outcomes in the four measurement times.

Group	Time	Outcome	Min	Max	Mean (SD)	Skewness	Kurtosis
Experimental (N = 27)	Pretest	FDS	61	106	81.85 (14.93)	0.359	−1.178
		TIBS	11	27	17.26 (3.89)	0.407	−0.187
		PCI	35	73	55.37 (10.61)	−0.142	−0.708
	Post-test	FDS	28	96	60.48 (18.80)	0.312	−0.711
		TIBS	10	24	16.59 (3.66)	0.251	−0.530
		PCI	24	71	51.78 (9.78)	−0.541	1.918
	3-month follow-up	FDS	28	81	58.52 (17)	−0.681	−0.738
		TIBS	6	25	15.48 (4.14)	−0.119	0.174
		PCI	31	70	50.59 (10.61)	−0.280	−0.571
	7-month follow-up	FDS	30	102	63.00 (17.59)	0.233	−0.291
		TIBS	6	24	15.48 (4.94)	−0.149	−0.608
		PCI	36	78	54.52 (10.59)	0.286	0.716
Control (N = 27)	Pretest	FDS	60	116	78.56 (13.49)	0.880	−0.828
		TIBS	9	23	16.85 (4.12)	−0.217	−0.634
		PCI	44	72	56.89 (7.50)	0.419	−0.754
	Post-test	FDS	44	99	77.11 (15.60)	−0.549	0.490
		TIBS	6	28	18.74 (4.64)	−0.669	0.259
		PCI	39	76	59.37 (8.26)	−0.506	1.430
	3-month follow-up	FDS	52	126	87.63 (17.27)	0.317	−0.200
		TIBS	10	26	18.59 (4.18)	−0.470	1.651
		PCI	43	93	58.63 (12.74)	1.311	−0.146
	7-month follow-up	FDS	52	102	74.30 (11.92)	0.319	−0.175
		TIBS	11	22	17.96 (2.98)	−0.667	1.716
		PCI	50	92	60.48 (10.50)	1.354	0.716

**Table 4:** Bootstrap analysis of the intervention’s total, direct, and indirect effects on pupil control ideology.

Effect	B	SE	LLCI	ULCI
Total effect	−7.59	2.45	−12.52	−2.65
Direct effect	−4.40	2.57	−9.57	0.756
Indirect effect	−3.18	1.71	−7.04	−0.390

## Data Availability

The data presented in this study are openly available in the OSF repository at https://doi.org/10.17605/OSF.IO/ZR84Y.

## References

[B1-ejihpe-15-00057] Abiogu G. C., Ede M. O., Agah J. J., Ebeh J. J., Ejionueme L. K., Asogwa E. T., Ekwueme F. O., Agu P., Nwafor B., Omeke F., Ogoke J. (2021). Effects of rational emotive behavior occupational intervention on perceptions of work value and ethical practices: Implications for educational policy makers. Journal of Rational-Emotive & Cognitive-Behavior Therapy.

[B2-ejihpe-15-00057] Ansley B. M., Houchins D. E., Varjas K., Roach A., Patterson D., Hendrick R. (2021). The impact of an online stress intervention on burnout and teacher efficacy. Teaching and Teacher Education.

[B3-ejihpe-15-00057] Baron R. M., Kenny D. A. (1986). The moderator–mediator variable distinction in social psychological research: Conceptual, strategic, and statistical considerations. Journal of Personality and Social Psychology.

[B4-ejihpe-15-00057] Bernard M. E. (1988). Teacher irrationality and teacher stress. 24th International Congress of Psychology.

[B5-ejihpe-15-00057] Bernard M. E. (2016). Teacher beliefs and stress. Journal of Rational-Emotive & Cognitive-Behavior Therapy.

[B6-ejihpe-15-00057] Bora C., Bernard M. E., Trip S., Decsei-Radu A., Chereji S. (2009). Teacher irrational belief scale-preliminary norms for Romanian population. Journal of Evidence-Based Psychotherapies.

[B7-ejihpe-15-00057] Bora C. H., Vernon A., Trip S. (2013). Effectiveness of a rational emotive behavior education program in reducing teachers’ emotional distress. Journal of Cognitive and Behavioral Psychotherapies.

[B8-ejihpe-15-00057] Caruso C., Angelone L., Abbate E., Ionni V., Biondi C., Di Agostino C., Mobili A., Verità R., Navarra R., Ruggiero G. M., Mezzaluna C. (2018). Effects of a REBT based training on children and teachers in primary school. Journal of Rational-Emotive & Cognitive-Behavior Therapy.

[B9-ejihpe-15-00057] Ciuhan G. C., Nicolau R. G., Iliescu D. (2022). Perceived stress and well-being in Romanian teachers during the COVID-19 pandemic: The intervening effects of job crafting and problem-focused coping. Psychology in the Schools.

[B10-ejihpe-15-00057] Clark L. (2002). SOS help for emotions: Managing anxiety, anger, and depression.

[B11-ejihpe-15-00057] Daniels L. M., Goegan L. D., Radil A. I., Dueck B. S. (2021). Supporting pre-service teachers’ motivation beliefs and approaches to instruction through an online intervention. British Journal of Educational Psychology.

[B12-ejihpe-15-00057] David D., Cotet C., Matu S., Mogoase C., Stefan S. (2018). 50 years of rational-emotive and cognitive-behavioral therapy: A systematic review and meta-analysis. Journal of Clinical Psychology.

[B13-ejihpe-15-00057] DiGiuseppe R., Doyle K., Dryden W., Backx W. (2013). A practitioner’s guide to rational-emotive therapy.

[B14-ejihpe-15-00057] Ding A. C., Wang H. H. (2018). Unpacking teacher candidates’ decision-making and justifications in dilemmatic spaces during the student teaching year. Asia-Pacific Journal of Teacher Education.

[B15-ejihpe-15-00057] Ellis A. (1962). Reason and emotion in psychotherapy.

[B16-ejihpe-15-00057] Ellis A., Dryden E. (1997). Group therapy in the practice of rational behavior therapy.

[B17-ejihpe-15-00057] Faul F., Erdfelder E., Lang A. G., Buchner A. (2007). G* Power 3: A flexible statistical power analysis program for the social, behavioral, and biomedical sciences. Behavior Research Methods.

[B18-ejihpe-15-00057] Gkontelos A., Vaiopoulou J., Stamovlasis D. (2023). Teachers’ innovative work behavior as a function of self-efficacy, burnout, and irrational beliefs: A structural equation model. European Journal of Investigation in Health, Psychology and Education.

[B19-ejihpe-15-00057] Guo L., Awiphan R., Wongpakaran T., Kanjanarat P., Wedding D. (2024). Social anxiety among middle-aged teachers in secondary education schools. European Journal of Investigation in Health, Psychology and Education.

[B20-ejihpe-15-00057] Harrington N. (2005). The frustration discomfort scale: Development and psychometric properties. Clinical Psychology & Psychotherapy: An International Journal of Theory & Practice.

[B21-ejihpe-15-00057] Harrington N. (2006). Frustration intolerance beliefs: Their relationship with depression, anxiety, and anger, in a clinical population. Cognitive Therapy and Research.

[B22-ejihpe-15-00057] Hayes A. F. (2022). Introduction to mediation, moderation, and conditional process analysis: A regression-based approach.

[B23-ejihpe-15-00057] Haynes A. B., Haukoos J. S., Dimick J. B. (2021). TREND reporting guidelines for nonrandomized/quasi-experimental study designs. JAMA Surgery.

[B24-ejihpe-15-00057] Huk O., Terjesen M. D., Cherkasova L. (2019). Predicting teacher burnout as a function of school characteristics and irrational beliefs. Psychology in the Schools.

[B25-ejihpe-15-00057] Knaus W. (1977). Rational emotive education. Theory into Practice.

[B26-ejihpe-15-00057] Knaus W. J. (1983). How to conquer your frustration.

[B27-ejihpe-15-00057] Lang S. N., Jeon L., Sproat E. B., Brothers B. E., Buettner C. K. (2020). Social emotional learning for teachers (SELF-T): A short-term, online intervention to increase early childhood educators’ resilience. Early Education and Development.

[B28-ejihpe-15-00057] Lazarus R. S., Folkman S. (1987). Transactional theory and research on emotions and coping. European Journal of Personality.

[B29-ejihpe-15-00057] Lupuleac C., Sava F. A. (2024). Romanian version of the Frustration Discomfort Scale (FDS): A preliminary validation on a non-clinical sample. Journal of Rational-Emotive & Cognitive-Behavior Therapy.

[B30-ejihpe-15-00057] Maag J. W. (2008). Rational—Emotive therapy to help teachers control their emotions and behavior when dealing with disagreeable students. Intervention in School and Clinic.

[B31-ejihpe-15-00057] Ogakwu N. V., Ede M. O., Manafa I. F., Okeke C. I., Onah S. O. (2024). Quality of work-life and stress management in a rural sample of primary school teachers: An intervention study. Journal of Rational-Emotive & Cognitive-Behavior Therapy.

[B32-ejihpe-15-00057] Onuigbo L. N., Eseadi C., Ugwoke S. C., Nwobi A. U., Anyanwu J. I., Okeke F. C., Agu P. U., Oboegbulem A. I., Chinweuba N. H., Agundu U.-V., Ololo K. O., Okpoko C., Nwankwor P. P., Eze U. N., Eze P. (2018). Effect of rational emotive behavior therapy on stress management and irrational beliefs of special education teachers in Nigerian elementary schools. Medicine.

[B33-ejihpe-15-00057] Panisoara I. O., Lazar I., Panisoara G., Chirca R., Ursu A. S. (2020). Motivation and continuance intention towards online instruction among teachers during the COVID-19 pandemic: The mediating effect of burnout and technostress. International Journal of Environmental Research and Public Health.

[B34-ejihpe-15-00057] Park D., Ramirez G. (2022). Frustration in the classroom: Causes and strategies to help teachers cope productively. Educational Psychology Review.

[B35-ejihpe-15-00057] Popov S., Popov B., Damjanovic R. (2015). The role of stressors at work and irrational beliefs in the prediction of teacher stress. Primjena Psihologija.

[B36-ejihpe-15-00057] Samfira E. M., Sava F. A. (2021). Cognitive-behavioral correlates of pupil control ideology. PLoS ONE.

[B37-ejihpe-15-00057] Samfira E. M., Sava F. A. (2023). The effectiveness of a rational-emotive intervention on teachers’ unconditional self-acceptance, perfectionism, and pupil control ideology. Frontiers in Psychology.

[B38-ejihpe-15-00057] Sava F. A. (2002). Causes and effects of teacher conflict-inducing attitudes towards pupils: A path analysis model. Teaching and Teacher Education.

[B39-ejihpe-15-00057] Shi S., Zhang Z., Wang Y., Yue H., Wang Z., Qian S. (2021). The relationship between college teachers’ frustration tolerance and academic performance. Frontiers in Psychology.

[B40-ejihpe-15-00057] Trip S., Vernon A., McMahon J. (2007). Effectiveness of rational-emotive education: A quantitative meta-analytical study. Journal of Evidence-Based Psychotherapies.

[B41-ejihpe-15-00057] Ugwoke S. C., Eseadi C., Onuigbo L. N., Aye E. N., Akaneme I. N., Oboegbulem A. I., Ezenwaji I. O., Nwobi A. U., Nwaubani O. O., Ezegbe B. N., Ede M. O., Orji C. T., Onuoha J. C., Onu E. A., Okeke F., Agu P., Omeje J. C., Omeke F., Ugwu R., Eneh A. (2018). A rational-emotive stress management intervention for reducing job burnout and dysfunctional distress among special education teachers: An effect study. Medicine.

[B42-ejihpe-15-00057] Warren J. M. (2013). School counselor consultation: Teachers’ experiences with rational emotive behavior therapy. Journal of Rational-Emotive & Cognitive-Behavior Therapy.

[B43-ejihpe-15-00057] Willower D. J., Eidell T. L., Hoy W. K. (1967). The school and pupil control ideology. Penn state studies monograph.

